# Ten Challenges for Systems Medicine

**DOI:** 10.3389/fgene.2012.00193

**Published:** 2012-09-27

**Authors:** Enrico Capobianco

**Affiliations:** ^1^Institute of Clinical Physiology (IFC), Laboratory for Integrative Systems Medicine (LISM), National Research Council (CNR)Pisa, Italy; ^2^Center for Computational Science (CCS), University of MiamiMiami, FL, USA

*Systems Medicine* (Auffray et al., 2009, 2010) emphasizes the role of systems biology in medical/clinical applications. With the advent of new technologies, the “omics” explosion (i.e., next generation sequencing) and the induced changes from data-poor to data-rich applications (for instance related to high-content imaging, physiology, and structural biology) have established the necessity of a systems approach (Noble, 2008) not to be caught in the data deluge. The accumulation and variety of high-throughput evidences and studies have generated hypothesis-driven models and validations at a previously inconceivable scale. Correspondingly, the assembly of models tackling all the implied complexities suggests challenges for which no standard (e.g., specified according to assumptions) inference approaches currently exist. In response to such problems and uncertainties, both data-driven intensive applications and model-free or agnostic (non-parametric) inferences are re-defining bioinformatics/statistics pipelines and network model architectures. Computational tools will be designed to satisfy criteria of: (1) *Efficiency* in processing, mining, and analyzing sequencing data; in particular, parallel architectures and high performance computing will be necessary to address the current data volumes and complexities; (2) *Flexibility* in synergizing the “omics” fields with clinical, biological, and environmental information whose integrative nature will require network-centric knowledge representation systems (Pawson and Linding, 2008; Zanzoni et al., 2008; Barabasi et al., 2011) will be very important; (3) *Accuracy* in data post-processing by exploiting model checking through robust feature selection and accurate output annotation, including clinical samples and patients’ follow up information.

The tasks required to satisfy such criteria are highly specific and technical, but show interrelationships that lead to systems approaches. From one hand, the components that need to be considered in such systems have heterogeneous features due to sample diversity acquired at data-poor (patients) and data-rich (cellular, imaging) resolutions, and require normalization to exploit complementary evidences (experimental, clinical, epidemiological, computational, simulation-based) and measurements (quantitative, environmental, perturbation-based). From another hand, a consensus concerning data collection and annotation is needed for comparative evaluations and assessment of data consistencies among studies and experiments.

Systems medicine represents a mosaic of distinct and interconnected micro-systems allowing to infer the macro-systems dynamics and produce elements of synthesis such as signatures (Hood and Friend, 2011; Sung et al., 2012) and profiles originated by a variety of information sources and consequently characterized. For instance, disease networks have been discussed by Barabasi et al. (2011), while pathway analysis beyond “canonical pathways” (Califano et al., 2012) and conceived for monitoring and assessing the mechanisms of action of drugs by the identification of targets and biomarkers, could involve multiple differential conditions to evaluate responses at system’s level or at global network scale (protein–protein interaction, gene regulatory, microRNA-target etc.), including deviation from equilibrium and/or stability. In response to crucial bottlenecks in Systems Medicine, our contribution aims to point out *10 challenges* that are going to characterize the field, and for which Figure 1 provides an ensemble view.

**Figure 1:**
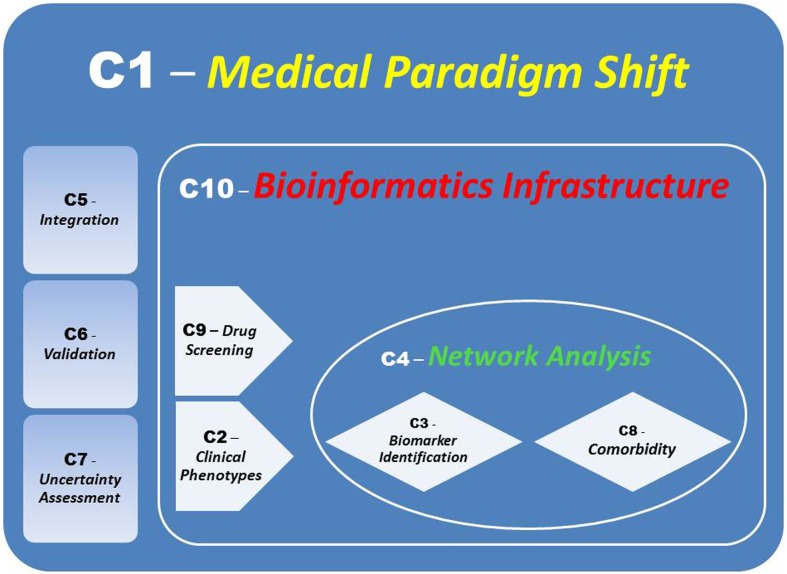
**Links between *Challenges***. A modularization of the *Bioinformatics Infrastructure* embedding integratively and significantly validated inferences will lead to a Systems Medicine Paradigm Shift.

## Shift of Medical Paradigms – How Does a Systems’ Approach Allow Translation to Effective Preventive & Personalized Medicine?

Cellular systems are globally organized entities consisting of interdependent components that contribute to the overall functionality and stability, and generate information from diversity, variation, and complexity achievable only synergistically, adaptively, and dynamically. Because of the combined interactions and effects between constituent entities, such systems embed prediction power available to discovery (target or biomarker identification) as well as inference (clustering and classification) in relation to pathological states of diseases, diagnostic or prognostic analysis, and preventive assessment (e.g., risk evaluation). The latter would prove the confluence of the system’s predictive power to patient-specific profiling (see Hood et al., 2012).

## Clinical Phenotypes – How Integrative Computational Models Re-Define Them?

New forms of multiplexed profiling will play a crucial role for representing and classifying phenotypic heterogeneity. They will address especially one of the current bottleneck in an attempt to make it a driving force: spatiotemporal high-dimensional profiles. Currently time and space dimensions are often poorly understood and overlooked in data modeling, with a loss of information. First, once analyzed in reduced dimensions and complexity they could be dynamically modeled at multiple scales (omics, clinics, imaging, computational) fused together. Second, their features (distributions, correlations, etc.) could be inferred by stochastic methods designed to deconvolute simply correlative or noisy patterns from causative information in time series. Stochastic systems allow for data-driven inference based on separation of signal from noise, and a more accurate assessment on the informative content of signals improves the definition and clinical impact of phenotype characteristics.

## Biomarker Identification – Are Biomarker Systems Useful to Represent and Interpret Complex Combinatorial Therapies?

Biomarker specificity induces differentiation that is only apparently contrasted at systems’ level. Substantial advantages with regard to individualized solutions related to tissue-specific studies and more effective diagnosis and therapy are emphasized by systems’ integrative aspects based on biomarker associations and dependencies that lead to combinatorial approaches. Biomarker networks may account for such complex observed and hidden relationships between genes and proteins explaining the combined and synergistic effects appearing at (sub-)modular scales. Consequently, panel biomarker studies may provide valuable functional inference and annotation quality to characterize both early diagnosis and response to treatment studies, thus justifying deviation from the commonly accepted biomarker models.

## Network Analysis – What is the Role of Networks in Disease Processing, and Their Potential for Elucidating Multiscale/Multilevel Inference?

Networks represent powerful flexible inference tools able to decipher very complex relationships between variables. In particular, when differential analysis (Ideker and Krogan, 2012) is performed to set comparisons between normal and disease states, or assess variation between disease states, measuring, and evaluating the changes, from global topological properties and modularity configurations to local intensity of network links, can establish the significance of such variation before conducting thorough validation. Variation can be accounted at topological level by the definition of metrics, based on vertex–vertex dependent distances or module-centric entropies, by the properties of modules (reduced dimensionality and complexity, specialization by tissue or cell type, etc.) that simplify inference, and by the consideration of time and space dynamics of disease processes.

## Integration – What Type of Integration Best Suits Systems Medicine Needs?

Integration allows to bypass the problem of sparsity of information due to partial or total lack of specific annotation in public repositories and biobanks. Part of the problems come from the need of associating heterogeneous entities retrieved from different sources: such multiple evidences potentially increase the overall system’s prediction power, but also propagate uncertainty of each source (experimental, clinical, etc.) to levels that might prevent from consistent statistical analysis. A first solution is finding normalizing and re-scaling transforms that allow the data diversity to be cast within a model framework. This way the source heterogeneity effects would be neutralized. A further step involves the control of the system’s redundancy in light of the degree of dependence of the integrated information. This way spurious and noisy correlations in data would be mitigated. These two actions would lead to a parsimonious normalized model with which to compute possible inferences.

## Validation – What is the Association between Computationally and Clinically Validated Evidences?

While clinical validation is always considered necessary, computational validation is often overlooked. Data-based results need to be tested for assessing the presence of real structural features instead of associations due just to chance. Statistical techniques and model checking strategies may determine significance in such regards. A more extended concept of validation involves replication strategies (montecarlo, bootstrap, bagging, etc.) and methodologies aimed to build unbiased “*null models*” that adapt to the nature of the data (see for interactome data Marras et al., 2010). Then, once benchmarks for complex data structures are found, computational validation requires testing over independent data sets to establish “*consistency*” of results.

## Uncertainty Assessment – How Reliable is Inference When an Integrative Dynamic Bio-System is Considered?

The confidence with which we assess the presence of informative dynamics instead of noise, or establish the presence of structural complexity instead of artifacts depends on factors such as the informative content of data, the model identifiability, the significance of results. Usually, sampling effects, errors, and experimental biases affect the accuracy of measurements at the experimental level. Limiting the effects of such aberrations involves the use of analysis too, through sampling and resampling techniques, intervention strategies for improving its robustness, and control of system’s stability. However, the role of noise and complexity must be deciphered and not simply neutralized or bypassed as they contribute to establish inference methods based on model robustness and regularization properties.

## Co-Morbidity – What Systems Re-Modularization is Expected after Shocks?

The equilibrium-disequilibrium dynamics involved in co-morbidity contexts (Bousquet et al., 2011) suggest that the effects of perturbing factors must be considered more carefully. For instance, when acute phases affect one or more pathological states, the co-morbidity map changes in two aspects: the role of each component in terms of relative relevance, and the interaction strength between components. The relevance of each component is determined in relation to the dominance exerted over the pathological patterns, and thus orienting the therapeutic decisions. The interaction strengths can be measured in terms of causative or propagation dynamics, and in relation to equilibrium versus non-equilibrium conditions. At system’s scale, a re-modularization involves the adaptive power of each constituent component in response to perturbation dynamics.

## Drug Development/Screening – Drug-Target and Drug–Drug Networks: What Future for Them?

Drugs directed to targets can be limited in their overall efficacy by the presence of network redundancies (off-targeted effects) and robust features (cross-talks) whose compensatory actions contrast or neutralize the target-related activities (Croft et al., 2011). Systems-oriented drug design improve our general understanding of the mechanisms of action, particularly when dealing with drug combinations and multi-target agents whose complex dynamics have just started to be studied (Goh et al., 2007; Jia et al., 2009). At systems’ level the power of network screening approaches refers to identifying phenotype varieties linked to the prediction of specific disease forms, and augmenting the druggable space through novel targets (Schadt et al., 2009).

## Bioinformatics Infrastructure – How to Effectively, Efficiently and Accurately Support and Perform the Translational Efforts Ahead?

By a wider access to a variety of information layers, an increased statistical power will be available from samples and their biological and clinical annotation for computational exploitation. An up-to-date bioinformatics environment would need to establish the fusion of all validated clinical, experimental, and computational evidences. In turn, multilevel knowledge management approaches (Szalma et al., 2010) will be required to overcome the infrastructural bottlenecks. In general, storage costs can hardly keep the pace of data-intensive bio-applications without technological developments. In particular, while high performance computing must address critical aspects involving replicated generations, parallel computations, and efficient storage, both analytical and algorithmic solutions will deal with complex inference tasks at multiple scales and together contribute to implement integrative research.
